# Reply to: ‘No direct evidence for the presence of Nubian Levallois technology and its association with Neanderthals at Shukbah Cave’

**DOI:** 10.1038/s41598-022-05049-6

**Published:** 2022-01-24

**Authors:** James Blinkhorn, Clément Zanolli, Tim Compton, Huw S. Groucutt, Eleanor M. Scerri, Lucile Crete, Chris Stringer, Michael D. Petraglia, Simon Blockley

**Affiliations:** 1 Pan-African Evolution Research Group, Max Planck Institute for the Science of Human History, Kahlaische Strasse 10, 07745 Jena, Germany; 2 Department of Geography, Centre for Quaternary Research, Royal Holloway University of London, Egham Hill, Egham, Surrey UK; 3 Univ. Bordeaux, CNRS, MCC, PACEA, UMR 5199, 33600 Pessac, France; 4 Department of Earth Sciences, Centre for Human Evolution Research, Natural History Museum, Cromwell Road, London, SW7 5BD UK; 5 Extreme Events Research Group, Max Planck Institutes for Chemical Ecology, The Science of Human History, and Biogeochemistry, Hans-Knöll-Strasse 8, 07745 Jena, Germany; 6 Department of Archaeology, Max Planck Institute for the Science of Human History, Kahlaische Strasse 10, 07745 Jena, Germany; 7 Institute of Prehistoric Archaeology, University of Cologne, 50931 Cologne, Germany; 8 Human Origins Program, Smithsonian Institution, Washington, D.C. 20560 USA; 9 School of Social Science, The University of Queensland, Brisbane, QLD 4072 Australia

**Keywords:** Cultural evolution, Archaeology

**replying to**: E. Hallinan et al.; *Scientific Reports* 10.1038/s41598-022-05072-7 (2022).

An exclusive connection between *Homo sapiens* and Nubian Levallois technology has been posited, but remains to be demonstrated^1^. Our re-evaluation of the fossil and lithic material from Shukbah Cave confounds such assumptions due to the identification of a Neanderthal molar tooth alongside Nubian Levallois cores and points at the site^2^. Hallinan and colleagues^3^ question this finding, instead supporting the use of Nubian Levallois technology as a *fossile directeur* to track expansions of *Homo sapiens*. We tackle these critiques, highlighting the problematic foundations in the assertion that Nubian Levallois technology is a unique, discrete entity, resulting in its misuse to support simplistic culture-historical narratives.

## Shukbah Layer D

Garrod’s excavation notes and publications on Shukbah indicate that finer stratigraphic diversity was recognised within the breccia deposits^4–6^, but both here and elsewhere it is evident that major sedimentary phases were resolved. The provenance of the Neanderthal molar and faunal records from the site both support a late Middle Palaeolithic timeframe, with no wider evidence from the site currently supporting substantive, earlier occupations. Garrod^4–6^ directly cautioned the rare presence of artefacts from distinctly later timeframes and younger technological systems within the Layer D assemblage may be attributed to the incised upper contact of Layer D, and is consistent with the 11 younger intrusive elements identified by Hallinan and colleagues^3^.

A late Middle Palaeolithic attribution of the assemblage is consistent with the typological character of the Layer D assemblage as reported by Garrod^4–6^, from a *chaîne opératoire* analysis by Callander^7^ that included the Rockefeller collection, and our attribute-based analysis of 707 artefacts^2^. Callander illustrates the uneven distribution of artefacts from Shukbah D between multiple major collections, with a notably higher proportion of retouched pieces and lower proportion of Levallois elements present in the small Rockefeller collection than elsewhere. We therefore suggest that the examination of a small and particularly biased sample by Hallinan and colleagues gives misleading insight into the character of the Shukbah D assemblage, as evident when contrasted with studies of much larger sample sizes by Garrod^4–6^, Callander^7^, and ourselves^2^. While we cannot preclude the possibility of earlier occupation of the site, the incidence rate of artefacts Hallinan and colleagues^3^ consider characteristic of such episodes (listed as LP [n = 3] or early MP [n = 7]) is extremely low (0.8%) amongst the artefacts recovered by Garrod (n = 1235), and may have been substantially lower had artefact retention practices been more comprehensive. Defining the presence of whole cultural phases based on single or rare artefacts, whilst overlooking the overwhelming majority of an assemblage, is questionable. On these terms, then, it would appear that any mixing of the Shukbah D assemblage has been minimal. The combined presence of a dominant focus on Levallois point production alongside centripetal and bidirectional flake production in the Shukbah D assemblage is directly comparable to other, dated, late Middle Palaeolithic sites, such as Kebara^8–10^, which were occupied by Neanderthals and contain comparable faunal records to Shukbah^6^. Assessments made by multiple researchers clearly demonstrate the classic late Middle Palaeolithic character of the Shukbah Layer D assemblage with a predominant focus on Levallois point production.

## The Neanderthal Molar Tooth

The provenance of the molar tooth is clearly described by Garrod^4–6^. Hallinan and colleagues^3^ present partial quotes from Garrod’s description to suggest that the Neanderthal molar derived from the contact between Layers D and B. Garrod unequivocally attributed the Neanderthal molar to the intact breccia deposits that are ascribed to Layer D: “A few human fragments were found in D, viz., a small portion of a temporal bone and a molar tooth, both from the base of the hummock on the edge of the pit”^6^. The tooth was overlain by ca. 2 m of Layer D breccias on the right side of the hummock with no direct contact with Layer B, *contra* Hallinan and colleagues^3^. Distinct stratigraphic contexts are described by Garrod for other human fossils potentially associated with abraded deposits^4–6^, which have no bearing on the secure attribution of the Neanderthal molar tooth to the Layer D breccias that yielded the late Middle Palaeolithic stone tool assemblage.

## Nubian Levallois technology

We documented nine cores in plan, as well as multiple views of two cores in SI Fig. 2 that visibly meet the four criteria set out by^11^, the parameters of which we used to identify the presence of Nubian Levallois technology in the Shukbah D assemblage. Hallinan and colleagues^3^ critique our predominant presentation of artefacts in plan (78%). Our approach is consistent with contemporary reporting of Nubian technology from Arabia by Usik and colleagues^11^, in the Levant by Goder-Goldberger and colleagues^12,13^, and in South Africa by Hallinan and Shaw^14^, where 65–95% of cores are only illustrated in plan. The broadest range of definitions of Nubian Levallois technology share focus on description of flaking surface scar patterns^1^, which are best observed in plan.

Hallinan and colleagues^3^ assert both the distinct form of Nubian Levallois reduction and a consensus in the criteria for its definition, following Usik and colleagues^11^. This overlooks considerable debate in the literature^1^. Usik and colleagues^11^ identify discrete modes of preparation of the flaking surface for Type 1, 1/2, and 2 Nubian Levallois cores, that Crassard and Hilbert^15^ suggest unnecessarily formalises plasticity, given that cores examined today are static elements of a past dynamic system. Goder-Goldberger and colleagues^12^ highlight that a definition that maintains the distinctness of Nubian technology is critical to its use as an “archaeological missing link”^12^, yet Usik and colleagues^11^ indicate that as distal flaking surfaces become flatter Nubian cores can be seen as “grading into bidirectional cores or recurrent cores”, Hallinan & Shaw^14^ identify cores with ‘Nubian affinity’ but not enough to confidently identify them as Nubian, and Rose and colleagues^16^ identify that “ there is overlap between Nubian Type 2 core preparation and some preferential point-producing Levallois reduction systems in the Levantine Mousterian”. Given this broad recognition that Nubian Levallois technology can grade into other Levallois approaches, some appraisal must be made of how and where to divide this spectrum of variability, as well as why.

No comparative basis to identify the categories chosen to define Nubian Levallois technology are set out by Usik and colleagues^11^, nor do they demonstrate them to be discrete from other Levallois methods. For instance, it is not established that there is a discrete distribution of distal flaking surface angle of Nubian and other Levallois cores that can be simply divided at 120 degrees, and in practice this quantitative threshold is exceeded elsewhere by Hallinan and co-authors^13,14^ in identification of Nubian cores. Nevertheless, to answer the Hallinan and colleagues^3^ critique the distal ridge angles on ten cores from Shukbah D stored at UCL have been measured. The angles range from 83° to 120°, with a mean of 102.4°,meaning that they are consistent with the particular descriptions set out by Usik and colleagues^11^ and favoured by Hallinan and colleagues^3^. Likewise, Hallinan and Shaw^14^ identify the difficulty in applying the shape categories, arguing instead for the need for quantitative analyses, which again should be established through comparative study if argued to be distinct features of Nubian Levallois technology. This remarkable absence of comparative studies prohibits acceptance that Nubian Levallois technology is inherently discrete from other Levallois methods.

Hallinan and colleagues^3^ contest whether Nubian Levallois end products can be identified, although they are commonly identified in the recent literature. For instance, Rose and colleagues indicate that Nubian Levallois reduction “results in the signature Nubian Levallois point”^16^, Goder-Goldberger and colleagues indicate “Other recognizable artifacts associated with the Nubian reduction sequence are the specific core shaping blades and the end-products”^12^ and that "the Nubian flaking system is unique and includes identifiable cores, preparation blanks and end products"^13^, whilst Hallinan and Shaw identify Nubian products from "various reduction strategies including Type 2, 1/2 and, to a lesser extent, Type 1 Nubian methods" from an appraisal of dorsal scar patterns^14^. Indeed, the need for quantitative study between Nubian and other Levallois points was highlighted as a "major limitation in the study of Nubian technology"^14^. Our identification of Nubian Levallois products is comparable to that set out by Hallinan and Shaw^14^ and provides a testable hypotheses for further analyses: that the use of Nubian Levallois reduction schemes has no significant impact upon the key metric attributes we studied in comparison to other Levallois points.

## Comparative Studies

We undertook extensive quantitative comparative studies of Late Pleistocene Middle Palaeolithic and Middle Stone Age toolkits across the Levant, Arabia and eastern Africa, a considerable research endeavour evident from the scarcity of similar analyses at comparable scope and scale. Indeed direct reappraisal of existing assemblages such as Aduma, rather than reliance on existing reports, is critical to evaluate diversity of Levallois reduction approaches; in this instance, we rejected previous assertions^17^ for the presence of Nubian Levallois technology. *Contra* Hallinan and colleagues^3^, who argue we overlook the potential role of raw material procurement on driving variability, we suggested that the immediate availability of larger clasts at the southern Arabian sites of TH123b and TH383 best explains the distinct variability observed in comparison with other sites, highlighting the larger size of these cores and the role of differential reduction intensity.

Nubian Levallois technology is best known from undated surface sites, typically occurring in low frequencies compared to other reduction methods. A narrow focus on Nubian Levallois technology has led to biased sampling practices that can result in the destruction of assemblage integrity (e.g.^13^), perhaps more so than Garrod’s excavation practices. These factors may have limited scope for comparative analyses to date but should similarly limit the use of this technology as a *fossil directeur* to track human expansions. Increasing sample sizes within and between sites will undoubtedly enhance precision of comparative studies, yet our results provide the means to test key hypotheses—whether Nubian Levallois is discrete from other Levallois technologies. Hallinan and colleagues^3^ argue that incorrect identification of Nubian technology best explains why considerable overlap exists between our Nubian and Other Levallois categories for Shukbah and other sites. However, this overlooks comparable results from other metric analyses^15^ as well as more extensive analyses involving refitting studies^18^, which support our results and demonstrate Nubian and ‘classic’ Levallois can be variants of a single technological approach.

## Conclusion

Amongst the plurality of definitions of Nubian Levallois technology in the literature, the basis upon which Usik and colleagues^11^ determined the criteria they set out is opaque, prohibiting clear differentiation from other Levallois methods. Hallinan and colleagues^3^ identify a core within the Shukbah D assemblage that meets key criteria set out by Usik and colleagues^11^. However as further ‘in-depth technological analyses’ are deemed necessary to resolve whether or not the core results from ‘a true Nubian reduction scheme’, it is apparent that these preferred criteria alone do not provide sufficient analytical utility to identify Nubian reduction schemes. This problem is exacerbated by Hallinan and colleagues^3^ asserting a single definition for the identification of Nubian Levallois cores, overlooking plurality in the literature, whilst simultaneously highlighting such plurality in previous studies as prohibiting the identification of Nubian Levallois point blanks. If, as Hallinan and colleagues^3^ assert, “a unified definition of Nubian technology is fundamental to a better understanding of its significance”, we consider it essential that such definitions are derived from comparative studies. In the absence of clear comparative studies that demonstrate the unique, discrete qualities of Nubian Levallois technology, we support suggestions that it forms part of a spectrum of variability amongst Levallois reduction approaches often associated with semi-arid landscapes^18^, which is consistent with its independent appearance in areas including southern Africa^19^. This provides the simplest explanation for its appearance at Shukbah, as well as the results of our comparative study. In contrast, the use of Nubian Levallois technology, and particularly its appearance in undated surface sites and the absence of robust association between artefacts and human fossils, to track the expansions of a discrete biological population, *Homo sapiens*, remains problematic. As a result, the use of Nubian Levallois technology by other populations that employ Levallois reduction methods and focus on the production of points is, perhaps, unsurprising.
